# Innovative Clinical-Organizational Model to Ensure Appropriateness and Quality in the Management of Medical Cannabis: An Italian Regional Case

**DOI:** 10.3390/healthcare9111425

**Published:** 2021-10-22

**Authors:** Eleonora Russo, Clara Cannas, Maria Susanna Rivetti, Carla Villa, Barbara Rebesco

**Affiliations:** 1 Section of Medicinal and Cosmetic Chemistry, Department of Pharmacy, University of Genova, 16132 Genova, Italy; villa@difar.unige.it; 2 Drug Policy Department, A.Li.Sa, 16121 Genova, Italy; clara.cannas@tiscali.it (C.C.); mariasusanna.rivetti@alisa.liguria.it (M.S.R.); Barbara.rebesco@regione.liguria.it (B.R.)

**Keywords:** medical cannabis, galenic preparations, standard operating protocols, monitoring data

## Abstract

This work focuses on the clinical-organizational model implemented in an Italian region (Liguria) to streamline the access procedures to galenic cannabis preparations. The competent local health care authority that takes care of tracing a virtuous path to obtain common, uniform and shared protocols and ensure high standards of care is A.Li.Sa. (Azienda Ligure Sanitaria), a public organization with the function of coordination, direction and governance of the health care in the regional hospitals and health facilities. To this purpose, different working groups and a board meeting have been set up with the main role to define and develop technical standards to be applied to the prescription, preparation and dispensing of pharmaceutical forms based on therapeutic cannabis. In particular, the galenic preparations provided by the Italian Ministry of Health, described in detail in the regional standard operating protocols, are described and discussed. Moreover, the most significant data monitored from 2018 to 2020 and collected by hospitals and the evaluation of those derived from local pharmacies and health facilities are presented, discussed and compared in regards to their adherence and coherence with the Italian Institute of Health (ISS) data.

## 1. Introduction

At the beginning of the 1900s, the United States was the first country starting a real prohibition of narcotic drugs. In 1912, the “International Opium Convention” was signed in The Hague [1] and in 1914, the Harrison Narcotics Act [2] restricted the sale of opiates and cocaine. Cannabis was removed from US Pharmacopoeia in 1942. In 1923, the US Treasury Department’s Narcotics Division banned the sale of all legal narcotics, including cannabis. In fact, in 1937 through the “Marihuana Tax Act” [3], the cultivation, trade and use of Indian hemp was banned. A medical interest in cannabis therapeutics arose in the 1940s when the American chemist Roger Adams chemically identified and synthesized cannabidiol (CBD), cannabinol (CBN) and several other molecules similar to tetrahydrocannabinol (THC), obtaining in 1942 a patent of the CBD isolation method [4,5]. In 1963, the Israeli chemist Raphael Mechoulam completely elucidated the properties and structure of THC, recalling and confirming Adams’ discovery [6]. Starting from the discovery of phytocannabinoids, the research turned toward the identification of the molecular pathways and receptor proteins involved in the signal transduction responsible for the numerous cannabis effects [7]. The discovery of cannabinoid receptors CB1 [8] and CB2 [9] dates to the 1990s when the receptor proteins, to which both exogenous and endogenous compounds were able to bind, were identified. At first, CB1 receptors were found in the brain, with the highest concentrations demonstrated in the basal ganglia, cerebellum, hippocampus, and cerebral cortex [10,11,12,13,14]; they were demonstrated to be involved in many brain functions such as movement, coordination, sensory perception, learning and memory, and emotions [15,16,17,18]. CB2 receptors occur in the periphery, like immune cells and the gastrointestinal tract [19,20,21], and in the central nervous system (CNS), mainly on microglia [22,23].

In Italy, with the first Law n. 396 of 18 February 1923 [24], the therapeutic use of narcotic drugs was allowed only for physicians and pharmacists for physicians (because of their profession). In 1961, the international agreement “Single Convention on Narcotic Drugs” approved by the United Nations and entered into force in 1975 gave further guidance on narcotic substances and drugs containing them. In Italy, the implementation of these regulations took place in 1975 with the Law n. 685 [25] unchanged until its revision, the Presidential Decree 309/90 [26], which is still in force. Since then, several more specific and applicable laws concerning import of medicinal specialties registered abroad [27], the regulation of magistral and officinal galenic formulations [28], and inclusion of cannabis-based preparations in Table II of Narcotic Drugs [29] were promulgated. With the last ministerial decree DM 9 November 2015 [30], Italy regulated the prescription of cannabis for medical use and the preparation of the relative pharmaceutical form performed by pharmacists (present in the attached technical annex to the law for the national production of substances and preparations of plant origin based on cannabis). Magistral galenic preparation is defined as the extemporaneous formulation that is set up in the pharmacy by the pharmacist following a medical prescription.

Under current legislation, medicinal use of *Cannabis sativa* L. may be administered orally in the form of decoction or cannabis oil extract as magistral galenic preparations. Decoction means the pharmaceutical form obtained by hot aqueous extraction from the cannabis inflorescences, contained in papers prepared by the pharmacist, while the oily product is obtained by hot extraction with olive oil (F.U. grade, Italian Official Pharmacopoeia).

As regards proprietary medicinal products, in Italy, the first cannabinoid-based pharmaceutical drug approved was Nabiximols (Sativex^®^, AIFA determination published in the Official Gazette n.100 of 30 April 2013). It is a standardized extract containing THC and CBD in a 1:1 ratio, which can deliver both the active ingredients through an oro-mucosal spray formulation. It is subject to a limited medical prescription drawn up by specific establishments (authorized by the regions) or by specialist physicians (i.e., neurologist). At first, the compilation of the AIFA log was planned, and then subsequently, (AIFA approval in Official Gazette n. 283 of 3 December 2019) starting from 4 December 22019, this monitoring log was abandoned. The drug was authorized for use by the National Health Service (NHS) in the control of spasticity in multiple sclerosis only when other treatments were not successful or excessive side effects occurred.

Other commercial products with standardized concentrations of THC and CBD are available, under the trade names of: “Bedrocan^®^” (22% THC and CBD < 1%), “Bedrobinol^®^” (13.5% THC and CBD < 1%), “Bediol^®^” (6% THC and 8% CBD), “Bedrolite^®^” (THC < 0.4% and 9% CBD) and “Bedica^®^” (14% THC and CBD < 1%), specifically produced by Bedrocan BV (The Netherland) on request from the Office of Medicinal Cannabis (BMC) of the Dutch Ministry of Health; “Pedanios^®^” variety (Pedanios 22/1, Pedanios 8/8, Pedanios 1/9) produced by Pedanios/Aurora (Canada) (see Table 1).

Starting from 2016, the “State Pilot Project for the national production of cannabis-based substances and preparations of plant origin” [31] started: it gave rise to two different types of cannabis products nationally and exclusively produced in the Stabilimento Chimico Farmaceutico Militare (SCFM) in Florence. The first type, introduced in December 2016, was called “cannabis FM2” (5–8% THC and 7.5–12% CBD) and the second, available from July 2018, was called “cannabis FM1” (13–20% THC and CBD < 1%).

In the Liguria region, the most widely used cannabis varieties for magistral galenic preparations were “Bedrocan^®^”, “Bediol^®^” and “Bedrolite^®^”, the Canadian cannabis “Pedanios 22/1” and the national cannabis products cannabis FM1 and cannabis FM2. In order to obtain greater coherence and efficiency in the medical cannabis dispensation and product tracking, all Italian regions have moved through local targeted projects aimed at simplifying, regulating and harmonizing any procedure related to cannabis therapeutics. This need arose from the beginning of the “National Pilot Project”, in which all technical specifications required by law had to be implemented, even if the national project was not successful.

Liguria in particular has organized, through the local health care authority A.Li.Sa., working tables and a board meeting made up by experts from different fields, for establishing shared technical standards and practices. All aspects related to the production and cultivation of the raw materials, the criteria to ensure the prescriptive appropriateness and dispensing, the treatment needs for which physician can prescribe these preparations for medical use, the pharmacodynamic and pharmacokinetic aspects, the side effects, the warnings and information on the risk of addiction, the phytovigilance system and, lastly, the costs of production have been considered.

Having taken part in the Ligurian board meeting of A.Li.Sa. on cannabis, as the expert and competent members of working groups in the pharmaceutical field, we aimed to describe all the activities, throughout the Ligurian region, carried out to standardize the management of cannabis magistral galenic preparations.

## 2. Materials and Methods

### 2.1. Working Groups

In order to achieve the objectives set, the local project started establishing multidisciplinary working groups that had to develop all health aspects related to the management of cannabis, providing the specifications necessary to ensure the support for all health operators and a strategy to gain compliance with the obligations under law. Considering that cannabis is a narcotic substance, it is mandatory to strictly comply with current legislation not only to avoid any risk in a disciplinary and administrative context but also to prevent criminal measures and sanctions.

The early working group was composed of physicians licensed in palliative/pain care, therapists within the network of pain management and public pharmacists with the task of:

- drawing up a strategic technical document aimed at assisting in the attainment of an updated daily working tool, capable of fostering transparency in the relationships between services, operators and patients, as well as being easily appliable at different operating realities.
- setting the rules, applicable by the Regional Health Service (SSR), for the delivery of galenic preparations, based on cannabinoids for medical use,
- ensuring the correct and safe use of these magistral galenic preparations.

Subsequently, a further group of professionals with specifically pharmaceutical expertise composed of pharmacists, professors of the Department of Pharmacy (DIFAR) of the University of Genoa and representatives’ figures of the scientific societies SIFO (Italian Society of Hospital Pharmacists) and SIFAP (Italian Society of Preparatory Pharmacists) was set up with the task of defining uniform operating procedures regarding the preparation of galenic formulations.

### 2.2. Regutalory Issues

The main useful instruments adopted by the working groups are legislative documents. In particular, the Italian Ministerial Decree D.M. 9 November 2015 [30] provided the technical specifications related to all aspects of cannabis (from cultivation, treatment and production of the raw material) and several phytovigilance documents for the supervision of side effects and drug interactions. The documents containing these significant data are provided by the Italian Institute of Health (ISS), the national competent health care authority responsible for monitoring cannabis safety. Its activity is made possible by the computerized VigiErbe phytovigilance system, which allows the collection, analysis and evaluation of the recorded reports in a specific online ISS portal. Anyone, doctors, health professionals and patients, by accessing this site can fill out card reporting forms regarding suspected adverse reactions. In particular, health professionals are required to report all suspected adverse reactions they observe during their institutional activity. The Italian legislation provides that regions transmit all relevant data related to patients treated with cannabis-based magistral galenic preparations to the ISS annually, complete with age and sex. This system was a very useful tool that allowed one to evaluate the significant epidemiological data and to investigate the real efficacy, safety profile and risk/benefit ratio concerning the use of cannabis preparations.

### 2.3. Drafting of Documents

In accordance with national measures, with Resolution no. 78/2018 [32], the working groups have drafted two technical-operational documents, the application of which is binding for the provision of such preparations by the Regional Health Service (SSR).

As regards the prescription, a document called “Recommendations for the prescription and management of magistral cannabinoid-based galenic preparations by the Ligurian SSR” has been elaborated to promote greater clarity in relations between services, operators and patients. This document establishes that Cannabinoid-based magistral prescriptions can be drawn up by any physician licensed by the Italian Medical Association. In the case of magistral preparations, where the costs are covered by the SSR, the prescription is only allowed for physicians licensed in palliative/pain care. A further constraint for the provision of these products, imposed by the SSR, consists in the compilation of the “Data Collection Form” or “Monitoring Form” on the ISS portal. In this sheet, the clinician has to report all the available information related to the treated patients: age, sex, dosage by weight of cannabis and the need for treatment, as well as the results obtained by the pathology treatment.

The other developed document has been called “A.Li.Sa. technical standards—magistral galenic preparations based on cannabis for medical use”. It defines the operating procedures for the procurement of the raw material and the achievement of the magistral galenic preparations based on cannabinoids: minimum structural and technological requirements are established in order to ensure the quality of the products; moreover, the detailed rules and good preparation standards allow one to achieve compliance and ensure the adoption of uniform procedures at the regional level.

The latest annex to Resolution no. 78/2018 “Summary of requirements for dispensing magistral galenic preparations based on cannabis for medical use by the SSR” [32] has established the responsibilities of different actors involved in the process from the prescription up to the delivery to the patient.

The most important and innovative aspect introduced by these regional protocols is to provide a critical and analytical regional overview regarding the monitoring of the prescription consumption and the treated patients in comparison with the clinical data proposed by the ISS.

### 2.4. Dissemination of Results and Diffusion of Information

To ensure maximum adherence to regional provisions, on 13 February 2019, the Board meeting has agreed to spread the information through a seminar, arranged with the clinicians of the “network of pain and palliative care”. During this, a shared procedure was defined for registering data in the aforementioned portal and how to compile the form was defined. This procedure has been formalized with a note by A.Li.Sa. n. 10,794 of 21 May 2019 [33].

### 2.5. Data Collection and Monitoring

The collected data are useful to verify the strength and reliability of the strategic approach from regional and national database sources. Every three months, each health facility (see Table 2) provides A.Li.Sa. (Health System of the Liguria Region), the central repository, the summary data related to the number of patients being treated and the analytical data distinguished by type of preparation provided. Consequently, from the ISS platform, the data referring to both the number of patients being treated and the number of prescriptions registered for each individual patient are extrapolated quarterly. These data are compared with those provided by each single Health Authority/Hospital/Facility.

The monitoring and analysis of the collected data represents a crucial point because in different contexts it allows one to ensure the therapeutic continuity of the patients under treatment and preserve the appropriateness and safety in use of the magistral galenic preparations (prescriptions and dispensations), but it also allows one to verify compliance with regional provisions. Finally, from the perspective of continuous improvement, it allows the control of the entire process, encouraging any improvement and/or corrective actions based on the information collected.

## 3. Results and Discussion

**Figure 1:**
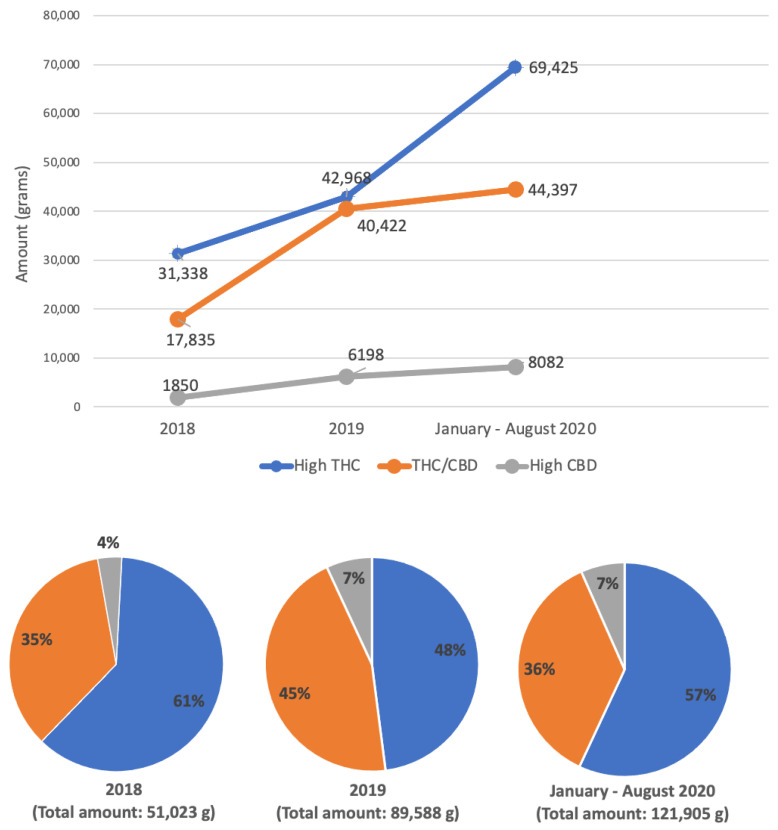
Regional summary: amount of purchased cannabis (in grams) and percentage distribution of different product types.

In order to obtain effective control on the availability of cannabis products, the monitoring of the supplied data for each individual health facility and hospital represent a crucial point. The most significant data are in regard to the detailed amount of purchased raw material and, above all, the measure of regional autonomy in terms of stock availability that ensures the monthly therapeutic coverage of patients under treatment [34]. The application of the correct legislative approaches and measures developed by the working groups has been monitored through this tool. Regarding this aspect, the details of the collected data are shown in Figure 1 and Table 2.

### 3.1. Acquired Data from Regional Monitoring (after the Entry into Force of the Regional Protocol)

Collected data have allowed one to confirm the strength and reliability of the regulatory approach obtained from the regional working groups. From the analysis of the numbers related to the Liguria region, it emerged that the quantity of cannabis purchased in the three periods considered (year 2018, year 2019 and January–August 2020) progressively increased, from a total amount of 51,023 g in 2018, to 89,588 g in 2019 up to 121,905 g in the 8 months of 2020 (Figure 1).

By going into detail, taking into account the different types of products in the three considered periods, the amounts of acquired cannabis were: 31,338 g (6%), 42,968 g (48%) and 69,425 g (57%) for cannabis with a high THC content; 17,835 g (35%), 40,422 g (45%) and 44,397 g (36%) for cannabis with similar THC and CBD content; 1850 g (4%), 6198 g (7%) and 8082 g (7%) for cannabis with high CBD content.

This procedure allowed the implementation of alternative strategies (e.g., possible loans between regional companies) in order to guarantee the therapeutic continuity of patients under treatment, even during difficulties in terms of product availability, due to raw material shortage. From the analysis of the collected data shown in Table 2, it is possible to detect the different prescriptive methods in quantitative terms of the various Ligurian health facilities.

In fact, the number of treated patients starting from 2018 until the third quarter of 2020 remained almost the same, about 1000 patients. Going into details, it can be noted that the largest number of treated patients, around half of the regional total, is situated in the ASL 2. The reason for this concentration is because a clinician, who strongly believes in the efficacy of these preparations in relieving the symptoms of patients, works in this area.

**Figure 2:**
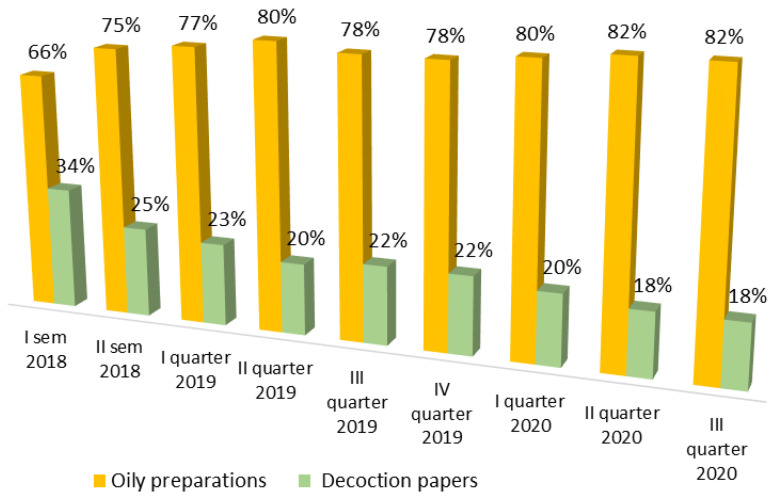
Distribution percentage of oily preparations and decoction papers, dispensed in Ligurian Region by the hospital pharmacist.

Two types of drug dispensing (decoction paper and extraction oil) are reported in Figure 2, which shows the percentage distribution of the formulations supplied in the Liguria region starting from the first half of 2018 until the third quarter of 2020. Over time, there has been a progressive shift in the types of pharmaceutical formulations supplied: in 2018, solid pharmaceutical formulations (papers, granulates) represented 34% of total deliveries; subsequently, there was a decrease in these products and a consequent, progressive, increase in oily liquid preparation until arriving, in the third quarter of 2020, to a value for the latter of 82%. This shift can be explained by the advantages of the oil: a greater simplicity of management that allows patients to take a more precise and accurate dosage of the active ingredients (THC and CBD), compared to solid preparations, and greater compliance by patients themselves.

Each oily preparation is in fact subjected to titration; this procedure quantifies the main active ingredients (THC, THCA, CBD, CBDA) contained in the preparations [35,36,37]. Unlike paper preparations and/or granules, it is not possible to trace their concentration, not only in the different dosage units, but also in the decoction that the patient makes for his/her own intake.

### 3.2. Adherence and Coherence of Regional Data in Comparison with National Data from the ISS Portal

Regional data were compared to the national data, which are registered on the ISS online portal. Table 3 shows the elaboration data related to treated patients and Table 4 shows results regarding the number of prescriptions. In Table 3, it is possible to verify that the values do not match.

As regards patients undergoing a treatment in the first quarter of 2019, the number on the portal was 625 with respect to 994 on treatment from the data provided by the Regional Health Authority (63% adherence); in the second quarter of the same year there was an improvement with 820 patients registered on the portal compared to the 1038 who were being treated by the data provided by the Regional Health Service (adherence equal to 79%) and, in the third quarter, there were 815 compared to 1037 (adherence equal to 78%). In 2020, the percentage of adherence decreased again, perhaps in consideration of the emergency period linked to the spread of the SARS-CoV2, which changed the priorities of healthcare professionals. The legislation provides that each prescription is valid for 30 days. The considered periods were quarters, therefore, on the ISS portal there should be three prescriptions for each patient. Going deeper into this aspect (see Table 4), an improvement at the regional level emerged. It went from an average value of 1.87 prescriptions for each patient in the first quarter of 2019, decreasing to 1.5 in the second quarter of 2019, up to an average of 2.1 prescriptions per patient in the second and third quarters of 2020.

## 4. Conclusions

In conclusion, the organizational process developed, through the sharing of uniform guidelines and procedures, led to the creation of a shared governance aimed at encouraging and guaranteeing equal access to cannabis-based magistral galenic preparations throughout the region. This strategy is aimed at ensuring quality, through compliance with the Good Preparation Standards (NBP), in terms of efficacy and safety of the product set up and supply. In addition, it was possible to activate and set up a system aimed at monitoring the process by defining the responsibilities of all health professionals involved. In particular, this system provides the central repository of results related to the supplying of raw materials, the prescriptions and dispensing of magistral galenic preparations in order to analyze and identify, through the systematic data collection, any critical issue and/or corrective actions to be undertaken with a view to continuous improvement, essential to the quality management process.

## Figures and Tables

**Table 1:** Commercially available cannabis products.

Producer	Name	CBD/THC Content
Bedrocan BV (The Netherlands)	Bedrocan	22% THC and CBD < 1%
	Bedrobinol	13.5% THC and CBD < 1%
	Bediol	6% THC and 8% CBD
	Bedrolite	THC < 0.4% and 9% CBD
	Bedica	14% THC and CBD < 1%
Pedanios/Aurora (Canada)	Pedanios 22/1	22% THC and CBD < 1%
	Pedanios 8/8	8% THC and 8% CBD
	Pedanios 1/9	THC < 1% and 9% CBD
SCFM (Italy)	FM2	5–8% THC and 7.5–12% CBD
	FM1	13–20% THC and CBD < 1%

**Table 2:** Patients under treatment with medical cannabis from 2018 to 2020.

	2018		2019				2020		
Health Facility	I Sem	II Sem	I Quarter	II Quarter	III Quarter	IV Quarter	I Quarter	II Quarter	III Quarter
San Martino *	27	55	40	46	48	66	85	95	88
Galliera *	6	24	28	21	21	17	17	19	19
Gaslini *	69	48	16	17	13	13	11	9	10
ASL1 **	57	75	75	88	82	85	95	88	79
ASL2 **	672	684	570	605	598	538	533	465	466
ASL3 **	65	59	56	41	71	91	101	109	106
ASL4 **	169	138	133	140	121	134	134	150	190
ASL5 **	105	82	76	80	83	83	95	129	104
Total	1170	1165	994	1038	1037	1027	1071	1064	1062

* Hospitals in the capital of the region; ** ASL is the acronym for Local Health Authority, the following number is indicative of a region area.

**Table 3:** Regional summary: number of treated patients.

Period	N. of Patients Registered on ISS Platform	N. of Patients (Data Provided by Regional Health Facility)	DELTA of Patients Number (ISS—Regional)	% Adherence to the ISS Portal
I quarter 2019	625	994	−369	63%
II quarter 2019	820	1038	−218	79%
III quarter 2019	815	1037	−222	78%
IV quarter 2019	707	1056	−349	67%
I quarter 2020	717	1071	−354	67%
II t quarter 2020	671	1064	−393	63%
III quarter 2020	671	1062	−391	63%

**Table 4:** Regional summary: number of prescriptions.

Period	Number of Medical Prescriptions	Number of Prescriptions on ISS Portal	Number of Patients on ISS Portal	Average Value Prescriptions/ Patient
I quarter 2019	22	1174	625	1.9
II quarter 2019	20	1231	820	1.5
III quarter 2019	21	1364	815	1.7
IV quarter 2019	20	1390	707	2.0
I quarter 2020	21	1414	717	2.0
II quarter 2020	21	1412	671	2.1
III quarter 2020	19	1412	671	2.1
Total	22	9397	5026	1.9

## Data Availability

The data presented in this study are available on request from the corresponding author. The data are not publicly available due to privacy.

## Abbreviations

A.Li.Sa	Ligurian Healthcare Company
AIFA	Italian drug agency
ASL	Local Health Authority
CB1	Cannabinoid receptors
CB2	Cannabinoid receptors
CBD	Cannabidiol
CBDA	Cannabidiolic acid
CBN	Cannabinol
CNS	Central Nervous System
DIFAR	Department of Pharmacy
DM	Ministerial Decree
DPR	Presidential Decree
IIS	Italian Institute of Health
FU	Italian Official Pharmacopoeia
NBP	Good preparation standards
NHS	National Health Service
SCFM	Military Pharmaceutical Chemical Industry
SIFAP	Italian Society of Preparatory Pharmacists
SIFO	Italian Society of Hospital Pharmacists
SRR	Regional Health Service
THC	Delta-9-tetrahydrocannabinol
THCA	Delta-9-tetrahydrocannabinolic acid

## References

[1] United Nations Treaty Collection International Opium Convention Signed at The Hague 1912, January 23, League of Nations Treaty Series, *8*, 188–239. https://treaties.un.org.

[2] Harrison Narcotics Act, Opium and Coca Leaves Trade Restrictions Act, in United States Statutes at Large, enacted by 63rd Unites States Congress 1914, Ch. 1, 38 Stat. 785. https://www.loc.gov/item/llsl-v38/.

[3] Marihuana Tax Act, Taxation of Marihuana, Enacted by the 75th United States Congress, 1937, August 2, 50 Stat. 551, Pub.L. 75–238. https://www.druglibrary.org/schaffer/hemp/taxact/mjtaxact.htm.

[4] Adams R., Hunt M., Clark J.H. (1940). Structure of Cannabidiol, a Product Isolated from the Marihuana Extract of Minnesota Wild Hemp. I. J. Am. Chem. Soc..

[5] Burstein S. (2015). Cannabidiol (CBD) and its analogs: A review of their effects on inflammation. Bioorg. Med. Chem..

[6] Michoulam R., Shvo Y., Hashish I. (1963). The structure of cannabidiol. Tetrahedron.

[7] Brunt T.M., Bossong M.G. (2020). The neuropharmacology of cannabinoid receptor ligands in central signaling pathways. Eur. J. Neurosci..

[8] Matsuda L.A., Lolait S.J., Brownstein M.J., Young A.C., Bonner T.I. (1990). Structure of cannabinoid receptor and functional expression of the cloned cDNA. Nature.

[9] Munro S., Thomas K.L., Abu-Shaar M. (1993). Molecular characterization of a peripheral receptor for cannabinoids. Nature.

[10] Glass M., Felder C.C. (1997). Concurrent stimulation of cannabinoid CB1 and dopamine D2 receptors augments cAMP accumulation in striatal neurons: Evidence for a G(s) linkage to the CB1 receptor. J. Neurosci..

[11] Hoffman A.F., Riegel A.C., Lupica C.R. (2003). Functional localization of cannabinoid receptors and endogenous cannabinoid production in distinct neuron populations of the hippocampus. Eur. J. Neurosci..

[12] Hohmann A.G., Herkenham M. (1999). Localization of central cannabinoid CB1 receptor messenger RNA in neuronal subpopulations of rat dorsal root ganglia: A double-label in situ hybridization study. Neuroscience.

[13] Mackie K. (2005). Distribution of cannabinoid receptors in the central and peripheral nervous system. Handb. Exp. Pharmacol..

[14] Wong D.F., Kuwabara H., Horti A.G., Raymont V., Brasic J., Guevara M., Ye W., Dannals R.F., Ravert H.T., Nandi A. (2010). Quantification of cerebral cannabinoid receptors subtype 1 (CB1) in healthy subjects and schizophrenia by the novel PET radioligand [11C] OMAR. NeuroImage.

[15] Bossong M., Jager G., Bhattacharyya S., Allen P. (2014). Acute and non-acute effects of cannabis on human memory function: A critical review of neuroimaging studies. Curr. Pharm. Des..

[16] Hill M.N., Hunter R.G., McEwen B.S. (2009). Chronic stress differentially regulates cannabinoid CB1 receptor binding in distinct hippocampal subfields. Eur. J. Pharmacol..

[17] Van Hell H.H., Jager G., Bossong M.G., Brouwer A., Jansma J.M., Zuurman L., Van Gerven J., Kahn R.S., Ramsey N.F. (2012). Involvement of the endocannabinoid system in reward processing in the human brain. Psychopharmacology.

[18] Zanettini C., Panlilio L.V., Alicki M., Goldberg S.R., Haller J., Yasar S. (2011). Effects of endocannabinoid system modulation on cognitive and emotional behavior. Front. Behav. Neurosci..

[19] Lombard C., Nagarkatti M., Nagarkatti P. (2007). CB2 cannabinoid receptor agonist, JWH-015, triggers apoptosis in immune cells: Potential role for CB2-selective ligands as immunosuppressive agents. Clin. Immunol..

[20] Lunn C.A., Reich E.P., Bober L. (2006). Targeting the CB2 receptor for immune modulation. Expert Opin. Ther. Targets.

[21] Wright K.L., Duncan M., Sharkey K.A. (2008). Cannabinoid CB 2 receptors in the gastrointestinal tract: A regulatory system in states of inflammation. Br. J. Pharmacol..

[22] Cabral G.A., Raborn E.S., Griffin L., Dennis J., Marciano-Cabral F. (2008). CB 2 receptors in the brain: Role in central immune function. Br. J. Pharmacol..

[23] Pertwee R.G. (2006). The pharmacology of cannabinoid receptors and their ligands: An overview. Int. J. Obes..

[24] Italian Law, L 396, 1923, Official Gazette (G.U. Serie Generale n.*53*, 05/03/1923). https://www.markab77.it/wp-content/uploads/2018/11/Gazzetta053_legge_396_1923.pdf.

[25] Italian Law, L 685, 1975, Official Gazette (G.U. Serie Generale n.*342*, 30/12/1975). https://www.gazzettaufficiale.it/eli/id/1975/12/30/075U0685/sg.

[26] Italian Law, DPR 309, 1990, Official Gazette (G.U. Serie Generale n.*255*, 31/10/1990—Suppl. Ordinario n. 67). https://www.gazzettaufficiale.it/eli/id/1990/10/31/090G0363/sg.

[27] Italian Law, DM 37, 1997, Official Gazette (G.U. Serie Generale n.*37*, 14/02/1997). https://www.gazzettaufficiale.it/eli/id/1997/03/05/097G0068/sg.

[28] Italian Law, L 94, 1998, Official Gazette (G.U. Serie Generale n.*86*, 14/04/1998). https://www.camera.it/parlam/leggi/98094l.htm.

[29] Italian Law, DM 23/01, 2013, Official Gazette (G.U. Serie Generale n.*33*, 08/02/2013). https://www.gazzettaufficiale.it/eli/gu/2013/02/08/33/sg/pdf.

[30] Italian Law, DM 9/11, 2015, Official Gazette (G.U. Serie Generale n.*279*, 30/11/2015). https://www.gazzettaufficiale.it/eli/id/2015/11/30/15A08888/sg;jses=.

[31] Pharmaceutical Chemical Military Facility in Florence. Medical Cannabis Production. 2019. https://www.farmaceuticomilitare.it/cannabis.aspx?lnrid=25.

[32] A.Li.Sa Aggiornamento Indirizzi per la Prescrizione, la Preparazione e la Somministrazione della Cannabis ad Uso Medico a Carico del SSR. Prot. Note n.78 of April 2018. https://www.sifoweb.it/images/pdf/attivita/sezioni-regionali/liguria/normativa_2018/Dlb_78-2018.pdf.

[33] A.Li.Sa Istruzione Operativa per la Compilazione Uniforme del Registro di Monitoraggio Dell’Iss per la Prescrizione della Cannabis ad Uso Terapeutico. Prot. Note n. 10794 of 21 May 2019. https://www.epicentro.iss.it/farmaci/pdf/Scheda%20prescrizioni%20cannabis.doc.

[34] A.Li.Sa Condivisione Indicazioni per Garantire L’accesso alle Preparazioni a Base di Cannabis. Prot. Note n. 17877 of 14 August 2019. https://www.alisa.liguria.it/index.php?option=com_docman&task=cat_view&gid=330&Itemid=609&limitstart=90.

[35] Baratta F., Simiele M., Pignata I., Ravetto Enri L., Torta R., De Luca A., Collino M., D’Avolio A., Brusa P. (2019). Development of Standard Operating Protocols for the Optimization of Cannabis-Based Formulations for Medical Purposes. Front. Pharmacol..

[36] Casiraghi A., Roda G., Casagni E., Cristina C., Musazzi U.M., Franzè S., Rocco P., Giuliani C., Fico G., Minghetti P. (2018). Extraction Method and Analysis of Cannabinoids in Cannabis Olive Oil Preparations. Planta Med..

[37] Palermiti A., Cafaro A., Barco S., Bucchioni P., Franceschini P., Cusato J., De Nicolò A., Manca A., De Vivo E.D., Russo E. (2021). Analysis of Cannabinoids Concentration in Cannabis Oil Galenic Preparations: Harmonization between Three Laboratories in Northern Italy. Pharmaceuticals.

